# Shape Modulation of Plasmonic Nanostructures by Unconventional Lithographic Technique

**DOI:** 10.3390/nano12030547

**Published:** 2022-02-05

**Authors:** Adriano Colombelli, Daniela Lospinoso, Roberto Rella, Maria Grazia Manera

**Affiliations:** CNR-IMM, Institute for Microelectronic and Microsystems, University Campus Ecotekne, Via per Monteroni, 73100 Lecce, Italy; daniela.lospinoso@le.imm.cnr.it (D.L.); roberto.rella@cnr.it (R.R.)

**Keywords:** colloidal lithography, localized and propagating plasmon resonance, metal nanohole array, nanoprisms

## Abstract

Conventional nano-sphere lithography techniques have been extended to the fabrication of highly periodic arrays of sub-wavelength nanoholes in a thin metal film. By combining the dry etching processes of self-assembled monolayers of polystyrene colloids with metal physical deposition, the complete transition from increasing size triangular nanoprism to hexagonally distributed nanoholes array onto thin metal film has been gradually explored. The investigated nano-structured materials exhibit interesting plasmonic properties which can be precisely modulated in a desired optical spectral region. An interesting approach based on optical absorbance measurements has been adopted for rapid and non-invasive inspections of the nano-sphere monolayer after the ion etching process. By enabling an indirect and accurate evaluation of colloid dimensions in a large area, this approach allows the low-cost and reproducible fabrication of plasmonic materials with specifically modulated optical properties suitable for many application in biosensing devices or Raman enhanced effects.

## 1. Introduction

In the last decades, an extensive interest towards plasmonic nano-structures has been established owing to their capability to confine and manipulate electromagnetic waves at the nanoscale level [1], as well as for their potential applications in several research fields, such as surface-enhanced spectroscopies [2,3], chemical or biosensors [4,5,6,7,8]. Innovative metamaterials characterized by exotic optical and physical properties have been proposed [9] for applications as ultrasensitive bio-chemical sensors exploiting surface plasmon resonance (SPR) properties [10].

Surface plasmons (SPs) are collective charge density oscillations that can be excited at a metal-dielectric interface [1,11]. Whether they occur as propagating waves at the metal-dielectric interface (known as surface plasmon polaritons (SPP)) [12], or as localized surface plasmon modes (LSPR) [13] close to metal nano-structures, SP excitation results in enhanced electromagnetic fields in the proximity of the metal structures, decaying exponentially into both the metal and dielectric medium [14]. SPPs experience a relatively deep penetration into the surrounding medium, creating large active volumes, while metal nanostructures supporting LSPR tend to tightly confine the enhanced electromagnetic field into subwavelength regions as small as a few nanometers. To this purpose, the variations in the refractive index of the surrounding external environment could be probed in a different manner, depending on the near field properties of the generated plasmonic field and taking into account also that the sensitivity parameters are strictly related to the spatial distribution of the electric field intensity near the metal surface [15], allowing the detection of biorecognition events occurring at its interface. However, plasmonic EM field amplification on a metal surface can be exploited for several applications like the enhancement of local signals in surface-enhanced spectroscopies, in photovoltaic energy harvesting [16,17,18,19], high-resolution microscopy [20], drug design [21,22,23,24,25], drug sensing [26,27] and nano-photonic devices [28,29].

The design and realization of plasmonic transducers with targeted functionalities still represent an interesting challenge. The aim of the present work is to exploit a low cost and reliable nanofabrication technique for the proper modulation of the plasmonic excitation on metal nanostructures arrays and the consequent high precision tuning of their spectral features. For instance, combination of different SP modes on plasmonic nanostructures arranged in periodic arrays can result in powerful variations of EM field intensity and distribution over the metal surfaces. More generally, engineering the geometrical properties of plasmonic nanostructures enables the control of their plasmon dispersion, influencing their spectral features, EM field localization, and consequently their functional abilities.

Although cutting-edge lithographic processes based focused ion beam milling (FIB) or electron beams (EBL) have successfully demonstrated their ability to control morphology and arrangements with high precision, the elevated cost of the equipment as well as the energy and time-consuming processes involved hinder their applicability in high-throughput devices. To overcome this issue, a growing number of low-cost and more accessible fabrication techniques have been recently developed. Nano-Sphere Lithography (NSL) is attracting considerable interest, owing to its potential to manufacture a wide variety of nano-structured materials as well as its compatibility with wafer-scale processes [30,31,32,33,34], providing a fascinating alternative to more expensive fabrication techniques. By depositing metals through the small apertures of a lithographic mask composed of a compact array of polystyrene (PS) nano-spheres, periodic arrays of gold nanoparticles with hexagonal distribution can be achieved [35,36,37,38].

In this context, our research group has matured its experience in the self-assembly of a close-packed array (CPA) of PS particles at air/water interfaces and their transfer on solid substrates. By developing a non-invasive, diffraction-based method to monitor the quality of PS monolayers, a high-quality, crystal-like nanostructure can be achieved. As-deposited PS masks were exploited first for the realization of an ordered arrangement of metal nanoprisms on different substrates. Our first attempts to tune their optical properties consisted of a reshaping of the as-deposited plasmonic structures by a controlled annealing process after metal deposition. The consequent spectral behavior and sensing abilities were demonstrated in two analytical contexts [39,40]. A different, still coarse approach focused our attention in the modification of the masks by a dry etching process [41,42,43] allowing us to realize of a non-close packed arrangement (NCPA) of PS masks. In that case, ordered nanohole arrays (NHA) in Au thin films [44] were achieved upon metal evaporation and mask removal.

Our research here intends to exploit the above-reported expertise in low-cost soft lithography to fine-tune the optical properties of metal nanostructure arrays at the nanoscale. Controllable nanometer-scale engineering of plasmonic resonance and EM field localization is a key factor in the realization of new functionalities or for enhancing surface-dependent optical phenomena [45,46,47,48,49]. Proper engineering of localized electric fields on the metal surface [44,50], can be exploited in different technological ambits: optical filters [51,52], the amplification of weak spectroscopy signals such as fluorescence [53,54] and Raman scattering [2,55,56], second-harmonic generation [57], and particularly molecular sensing [58].

In this paper, by combining a dry etching processes of PS colloid assemblies with metal physical deposition, further insights in the far- and near field properties of metal-arrayed nanostructures are opened. A sharp tuning of their size and geometry results in a variety of plasmonic features with different functionalities. This paper details how the tunability and suitable optical properties of these calibrated transducers enable their use as a multiprobe sensing system in the visible spectral range, by simply using a single optical transducer. Moreover, their integration into a miniaturized microfluidic platform allowed us to characterize their sensing performance in a liquid-controlled environment. Finally, as a proof of concept, the potential of the plasmonic transducer as a multiprobe detection system at different wavelengths was tested by refractive index measurements.

## 2. Materials and Methods

### 2.1. Fabrication of Close-Packed Arrays

**Figure 1:**
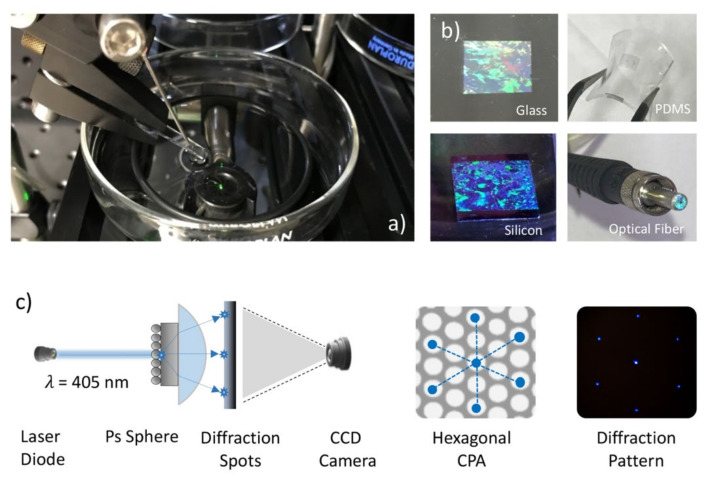
(**a**) Photograph of the setup for CPA self-assembly at the air-water interface; (**b**) image of the CPA monolayer after transference onto glass, PDMS, silicon and the small tip of an optical fiber; (**c**) schematic representation of the diffraction tool used for the inspection of CPA crystal quality.

The self-assembly of PS nanospheres was performed in a homemade setup, schematically reported in Figure 1a. Details about the experimental setup as well as the deposition process can be found in ref [40]. Briefly, a PS particle suspension was slowly dispensed by a motorized syringe pump onto a tilted glass slide partially immersed in a water-filled Petri dish. Polystyrene particles with a nominal diameter of 500 nm were used in the experiments; the particles were purchased from Sigma-Aldrich in aqueous suspensions with a concentration of 10 wt% and density of 1.05 g/cm. In order to trap the polystyrene nanospheres at the air/water interface, a mixture of alcohol and polystyrene water solution was employed. In this work, the polystyrene particle solution was diluted to a final concentration of 2.5 wt% in a 1:1 ratio with ethanol, which acted as a spreading agent on the water surface. After the close-packed array’s formation at the air/water interface, the colloidal mask can be deposited onto a solid or a flexible substrate, as shown in Figure 1b, where several CPAs of nano-spheres with a diameter of 500nm have been transferred to glass or silicon flat substrates, onto a flexible PDMS support, and onto the small tip of an optical fiber.

### 2.2. Fabrication of Non-Close Packed Array

**Figure 2:**
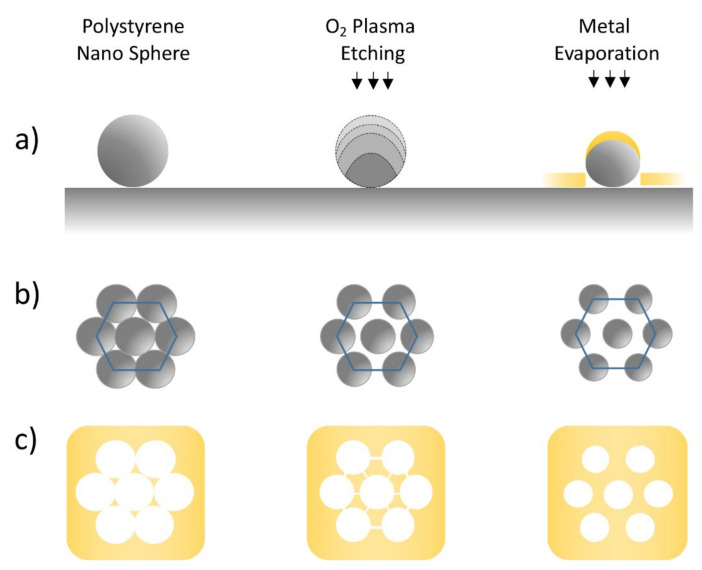
(**a**) Schematic illustration of the NHA fabrication process. From left to right, the main three steps are, respectively: PS nanosphere deposition onto solid substrate, sphere size reduction through oxygen plasma treatment, and metal layer evaporation. (**b**) The progressive reduction of NS size induced by oxygen plasma treatments and (**c**) the results obtained after the metal deposition and = lift-off process.

A post-deposition process based on oxygen plasma etching can be applied on the colloidal mask in order to induce a progressive reduction of the sphere size and the generation of a non-close packed array (NCPA) of PS nanospheres, as schematically reported in Figure 2a. The polystyrene spheres’ diameter can be precisely reduced to the desired dimension while their periodicity remains unchanged within all masked areas (Figure 2b). The etching process has been investigated on different kinds of substrates by using a plasma cleaning setup (Diener ATTO, Diener electronics, Ebhausen, Germany). Polystyrene spheres were etched using 40 W RF (radiofrequency) power plasma at an oxygen pressure of 0.25 mbar, and variable exposure times that depended on the initial sphere size and the desired size reduction. In order to obtain a reproducible plasma atmosphere, a stabilization process of 200 s was carried out for the vacuum pump and the gas flow. To prevent the temperature from increasing inside the etching chamber and the possible melting of the PS nanospheres, the etching and cooling steps were alternated during the plasma treatment. Regardless of the total treatment time, the plasma was switched off after 120 s of exposure, allowing the chamber to cool down for at least 60 s before starting the subsequent step of the treatment. The reshaped polystyrene nanosphere mask enabled the fabrication of a periodic array of different kinds of metal nanostructures, from metal nano prisms with a triangular shape and increasing size to hexagonally distributed nanoholes array on a thin metal film (Figure 2c).

### 2.3. Morphological and Optical Characterization

The morphology of a typical fabricated colloidal mask deposited onto glass substrates was investigated by Atomic Force Microscopy (AFM NT-MDT Spectralight Moscow, Russia, operating in semicontact mode, with Si tips) before and after the oxygen plasma treatment. Similarly, the morphology and size of the gold nanostructures obtained were characterized by the same AFM facility. The optical properties of the colloidal mask deposited onto glass substrates and the plasmonic properties of the realized Au nanostructures were characterized in the Vis-IR spectral range using a Cary500 UV-VIS-NIR spectrophotometer (Varian, Palo Alto, CA, USA).

The functional characterization of the fabricated plasmonic transducers was evaluated, in the transmission configuration and liquid phases by using a compact optical fiber system described in [39]. This portable system was equipped with a portable spectrophotometer (USB2000 UV–Vis, wavelength ranging between 250 and 1100 nm, Ocean Optics, Oxford, UK), a tungsten halogen light source (LS-1, wavelength range 360–2000 nm, Ocean Optics) and two optical fiber probes (R-400-7 UV-VIS, Ocean Optics). The white light emerging from the optical fiber was vertically focused onto the nano-structured surface of the sample at room temperature. The transmitted light was analyzed in the UV-VIS spectral range using a portable spectrophotometer. In order to perform a refractometric test, the spectral shift of the plasmonic signal was investigated. The typical LSPR absorption peak, or transmittance minimum, was followed in presence of an external environment characterized by an increasing refractive index. Absorbance and transmittance measurements were performed in air (n = 1.00), water (n = 1.33), and different water/glycerol solutions. By increasing the glycerol concentrations, the response of the transducer was tested in a selected range of refractive indices ranging between 1.33 (pure water) to 1.47 (pure glycerol).

### 2.4. Colloidal Mask Characterization

The formation of a crystal-like structure on the air-water interface can be directly observed during the self-assembling process. Under white light illumination, structural colors appear, as can be noticed in Figure 1b. This effect arises from the Bragg reflection of light by periodic structures [9]. Furthermore, a monitoring technique based on laser diffraction schematically reported in Figure 1c has been developed to investigate the quality of the colloidal mask on transparent substrates. A blue laser diode with a wavelength of 405 nm was used to investigate lattice quality and orientation after the transfer to a solid substrate.

The significant variations of nano-particle dimensions induced by the oxygen plasma treatment deeply affect their optical properties. This effect can be easily detected by monitoring the color changes of the colloidal mask. Therefore, to find a close relationship between the size of the reduced nano-spheres and the optical properties of the etched colloidal mask, absorbance measurements were performed after each step of the etching process.

### 2.5. Nanohole Array Fabrication

The above-described process induces the formation of non-close packed arrays (NCPA) of nanospheres characterized by the desired dimension. The NCPA of PS nano-spheres can be exploited for the fabrication of long-range ordered nanohole (NH) arrays realized into a thin metal film [59]. The holes’ periodicity is strictly related to the geometrical parameters of the colloidal mask, depending specifically on the diameter of the chosen PS nano-sphere. The holes’ diameter, instead, can be precisely modulated by varying the duration of the oxygen plasma treatment [42].

Standard Electron Beam Evaporation (EBE) was adopted for the nano-structure fabrication. In particular, a layer of gold with a nominal thickness of 30 nm was deposited on the mask, allowing the formation of nanoholes array with a periodicity of 500 nm. A 2 nm thick titanium layer was used as an adhesion layer on the glass surface.

By removing the colloidal mask with a chemical or mechanical lift-off process, the fabricated nanostructures can be revealed. Mechanical stripping of the PS nano-spheres by an adhesive tape can be performed on particles with larger diameters. However, a chemical approach is preferred with smaller particles to avoid nonuniform removal of the colloidal mask. An ultrasonication step in toluene was performed for several hours to safely remove PS nano-spheres smaller than 200 nm.

## 3. Results

**Figure 3:**
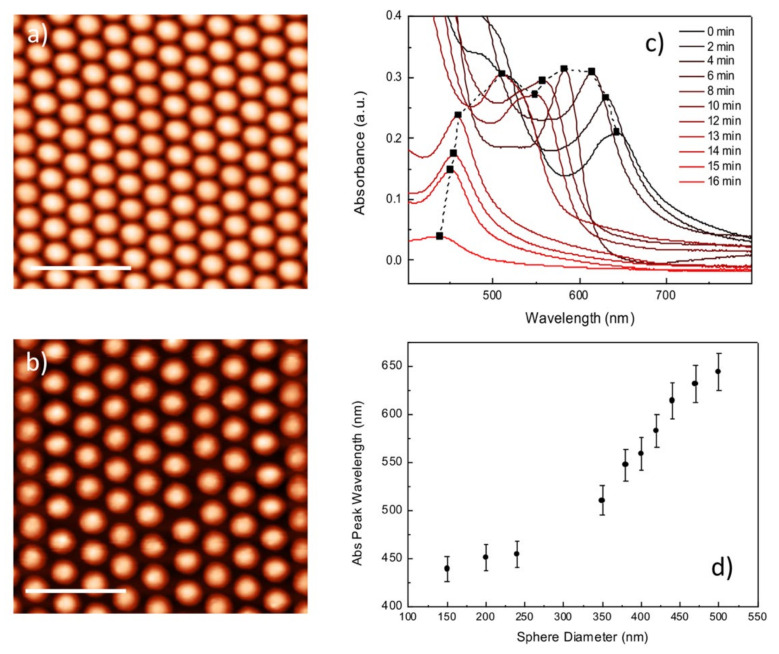
(**a**) AFM images of the CPA of PS nano-spheres deposited onto a glass substrate and (**b**) of the NCPA obtained after plasma etching; the scale bar is 2 μm;.(**c**) Evolution of the absorbance spectra, upon oxygen plasma treatment, for the as-deposited and etched PS nano-sphere arrays. (**d**) The spectral position of the absorbance peak is plotted as function of the measured nano-sphere dimensions.

Recently, several groups investigated the self-assembly of colloidal particles at the air/water interface [59]. The home-made experimental setup and the deposition process exploited in this work have been extensively described by our group in ref [40]. Based on a fine balance between different physical forces, including inter-particle electrostatic forces and flotation capillary forces, this process enables the fabrication of monolayers of PS nano-spheres with a diameter of 500 nm onto solid and flexible substrates, as can be seen in Figure 1b. By perpendicular irradiation, with a blue laser, of the CPA deposited onto a transparent substrate, a diffraction pattern characterized by hexagonal geometry can be generated. By using an appropriate hemispherical lens, the diffracted light beams can be projected onto a paper screen and detected in real-time using a CCD camera, as can be seen in Figure 1c. The crystal quality, as well as the lattice orientation, can be easily determined from these relative diffraction patterns. In fact, a clear hexagonal diffraction pattern should be observed with well-defined maxima, suggesting the presence of long-range structural ordering in the area illuminated by the laser beam. Figure 3a shows AFM images of the assembled monolayer of PS nano-spheres with an average diameter of 500 nm. As one can see, a uniform close-packed array of PS nano-spheres can be achieved using the deposition technique described above with long-range order and few lattice defects.

The size and distribution of the PS nanospheres starting from close-packed configuration towards the formation of NCPA were precisely controlled by exploiting appropriate oxygen plasma treatments. Oxygen plasma etching induced the controlled shrinkage of individual PS nano-spheres without displacing them from their hexagonal periodic distribution. This is shown in Figure 3b, where a pristine colloidal mask with 500 nm PS particles (Figure 3a) was etched for 12 min, resulting in a hexagonal NCP array of PS nano-spheres. The etched arrays were very well spaced and uniform, showing a remarkable control over the nano-sphere dimensions. The complete transition from increasing-size triangular nanoprisms to hexagonally distributed nanoholes array thin metal film were gradually explored by increasing the O_2_ etching time. O_2_ plasma etching was carried out from 2 to 16 min to progressively reduce the diameter of the PS nano-spheres. The particles’ geometry after each dry etching step, was investigated with AFM measurements, and the corresponding diameters arereported in Table 1.

The optical property modifications induced by the etching process were also monitored by the absorbance spectra recorded in the UV-VIS NIR spectral range. As can be noticed in Figure 3c, the dry etching process induces an evident blue shift in the absorbance peak of the PS monolayer deposited onto a glass substrate. Such effects become more evident as the etching time increases, underling the close relationship between the dimension of the spheres and their optical properties. Exploiting the results obtained from morphological AFM analysis, a relation between the diameter of the nano-spheres and their optical absorbance peak was found and reported in Figure 3d. This optical method can be easily implemented during the fabrication process to dynamically monitor nano-sphere size in a very large area. The diameter of the spheres can be reduced on-demand in a controllable way to the desired size, allowing the very fast and reproducible nano-structured plasmonic material fabrication over a large area (centimeters square) with a low-cost lithographic technique.

**Figure 4:**
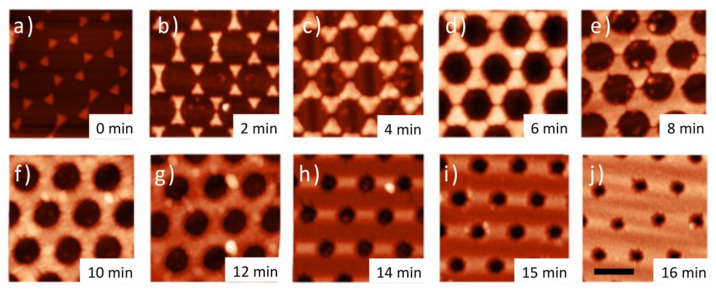
(**a**) High-resolution AFM images of gold nanostructures obtained from the (**a**) as-deposited CPA and (**b**–**j**) the etched NCPA, after metal deposition and sphere removal. The samples were obtained with increasing etching time of the colloidal mask. The corresponding treatment times were (**b**) 2, (**c**) 4, (**d**) 6, (**e**) 8, (**f**) 10, (**g**) 12, (**h**) 14, (**i**) 15, and (**j**) 16 min; the scale bar is 500 nm.

A two-step metal evaporation was performed starting from the deposition of a 2 nm thick Ti, layer followed by an Au layer with a nominal thickness of 30 nm. The colloidal mask lift-off revealed the realized nanostructures. As can be seen in the AFM images reproduced in Figure 4, the progressive reduction of the PS nano-spheres by increasing time of O_2_ plasma treatment allowed the exploration of the complete geometrical transition from hexagonally distributed triangular nanoprisms towards well-defined nanoholes array on thin metal film. As can be noticed in Figure 4a, unetched colloidal templates enabled the formation of periodic nanoprisms characterized by triangular shapes and very sharp tips. A short O_2_ plasma treatment induced the formation of nano-bridges, along with the contact points of the individual PS particles which have been previously reported in the literature [60]. Often, these polymeric inter-particle connections are not uniform in size and distribution, which makes them undesired defects, rather than precisely nanoengineered features. However, these nano-bridged colloidal templates can be exploited for the fabrication of metal nanostructures with more complex triangular shapes and rounded edges, as can be noticed in Figure 4b,c. Further oxygen plasma treatment gradually shrinks the PS nano-spheres and thins the inter-particle connections, resulting in the fabrication of metal nanostructures of variable size and shape. In detail, structures with a larger triangular shape, circular nanoholes array in metal film, or even a transition stage between the two can be produced (Figure 4d,e).

The PS sphere diameters were further reduced by an additional etching process, inducing the formation of a NCPA of particles. In this configuration, the fabrication onto the thin metal film of hexagonally distributed NH characterized by decreasing diameters was obtained (Figure 4f,j). The smallest NH diameters were achieved by processing the colloidal mask for a total time of 16 min. A final diameter of 150 nm was achieved, reducing the pristine PS particles’ dimensions by at least 60% in size, without losing their periodic distribution.

An interesting and reproducible relationship between the oxygen etching time, PS nano-sphere size and their optical absorbance properties was found, as reported in Figure 3d. The resulting colloid dimensions can be tailored with very high precision to produce diameters ranging between 500 to 150 nm in size. This result is essential for the rapid manufacture of plasmonic metamaterial on a very large area with optical features specifically tuned in the Vis-IR spectral range.

**Figure 5:**
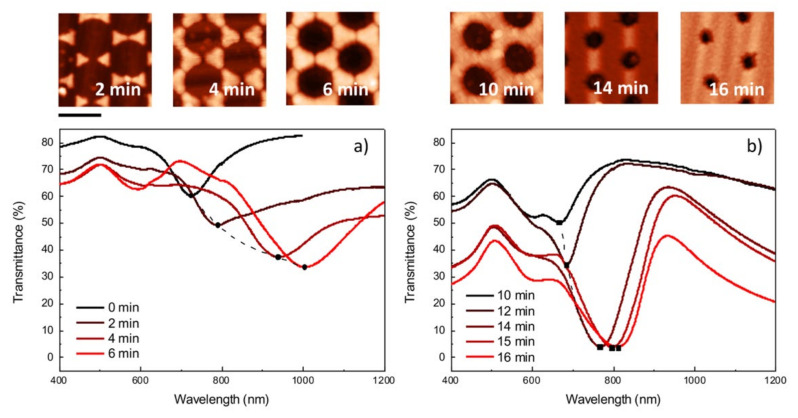
Evolution of the optical transmittance spectra upon oxygen plasma treatment of the colloidal mask; (**a**) a short O_2_ treatment (2–6 min) induces a red shift of the spectrum, linked with the formation of larger triangular nanoprism; (**b**) as the etching time increases (8–16 min) a gradual transition from isolated nanostructures to hexagonally distributed nanoholes array can be induced, generating considerable variation of the optical properties. Examples of the metal nanostructures obtainable with short or long etching time have been reported in the high-resolution AFM images above each graph.

The optical characterization of the fabricated plasmonic surfaces is reported in Figure 5a,b, showing the transmittance spectra in the 400–1200 nm spectral range. In the case of the sample with zero-minute treatment, the related spectrum was acquired in the range of 400–900 nm. The oxygen etching time needed for the fabrication of each sample was chosen for its identification in this graph and is easily associated with its respective AFM image (Figure 4). Due to the excitation of the localized or propagating SPR, the plasmonic signals of the fabricated materials exhibit interesting optical features in the visible and near-IR spectral ranges. A periodic array of triangular nanoprisms, realized by using unetched colloidal templates, exhibits a pronounced dip in transmission around λ = 726 nm. The dip can be attributed to the ensemble average dipolar plasmon resonance of the Au nanoprism arrangement [35].

Short O_2_ plasma treatment on colloidal templates induces significant variations to both the morphological and optical properties of the fabricated nanostructures. The evolution of the optical transmittance spectra upon oxygen plasma treatment is reported in Figure 5a. A short etching process (2, 4, and 6 min) induces an evident red shift in the spectrum (evidenced by the dotted line in Figure 5a), accompanied by a clear broadening of the band: from 726 nm with the 0 min sample to 791 nm, 940 nm and 1005 nm with the 2 min, 4 min and 6 min samples, respectively. As can be noticed in Figure 5a, this effect results in the formation of larger nanoprisms with additional edges and increasingly small inter-distances. A decrease and redshift in the transmission minimum is the effect recorded in the far field as a consequence of an increasing radiation damping as metal structure size grows up [2,36]. Plasmonic hot spots are expected to occur in the small spaces between nanostructure assemblies where the EM field of the incident light becomes strongly enhanced. The consequent increased optical absorption can be then released radiatively or non-radiatively, causing valuable physical effects. Surface-enhanced phenomena such as Raman scattering can benefit from the radiative decay channel, while heat generation or hot plasmonic electron excitation could be a consequence of the second decaying route. Relying more on the intrinsic quantum properties of the excited Fermi gas in metal nanocrystals, the generation of electron–hole pairs can be studied when adsorbate molecules or semiconductors are attached to the metal nanostructures. In this case, hot electrons can be harvested and extracted to the adsorbate or a semiconductor before thermalization into heat. The phenomenon has great interest in the scientific community, as it can be harnessed for photovoltaics, photocatalysis, and photodetection as well as other electrochemical processes such as water splitting [61,62,63,64].

Investigations of the above applicative aspects are out of the scope of the paper. It is worth noting that the effective aim, namely the design and realization of a plasmonic nanocrystal assembly with tunable optical properties, is of crucial importance for the creation of hotspots with different functional abilities and consequent applicative ambits.

As the time of the etching process increases, the gradual transition from isolated nanostructures to hexagonally distributed nanoholes array in continuous gold film determines the appearance of a completely different regime with distinctive optical properties (Figure 5b). With increases to the mask etching time the transmission maximum at higher wavelength appears sharper. As the NH diameters decrease, the transmittance minimum undergoes a clear red-shift towards the IR spectral range: this behavior can be explained by invoking the Babinet principle, which establishes a correspondence between the reflectance of a nano-disk array and the transmittance of its complementary NH array [37]. For the samples treated for 10 and 12 min, the dipolar LSPR of the just-formed NHs is expected to be in the infrared region, and thus it is not visible in the investigated region. Instead, samples treated for 14, 15 and 16 min can be identified by the transmittance dips located at about 768, 800 and 810 nm, respectively.

Moreover, it is possible to observe the presence of additional weak maxima and minima at lower wavelengths. This variegated pattern of optical transmittance measurements in the Vis-IR spectral range is linked with hybrid plasmonic modes due to the simultaneous excitation of both the localized and propagating SPR inside this kind of nano-structured thin metal film. In the case of nanohole arrays, their grating-like nature provides the extra momentum needed for free-space coupling and the formation of surface plasmon polaritons (SPPs). Peaks in the transmittance spectra of nanoholes array in metal films are usually related to the enhanced transmission phenomenon known as extraordinary optical transmission (EOT) through subwavelength apertures in a metal film [65]. This phenomenon occurs when the SPP Bloch Wave (SPP-BW) modes on the two sides of a hole array interact, re-radiating the incident field and increasing the optical transmission [66,67]. For hexagonal arrays of nanoholes, the resonance condition can be written as [68]:

(1) $$\lambda_{SPP}\left(i,j\right)=\frac{\sqrt{3}}{2}\frac{P}{\sqrt{i^{2}+ij+j^{2}}}\sqrt{\frac{\varepsilon_{m}\varepsilon_{d}}{\varepsilon_{m}+\varepsilon_{d}}}\;$$

where $\lambda_{SPP}$ represents the SPP resonant wavelength, P is the lattice spacing of the NH array, i and j are integer indexes of the peaks which represent the Bragg resonance orders of the array, and $\varepsilon_{m}$ and $\varepsilon_{d}$ are the real parts of the relative permittivity of the investigated material (gold in our case) and the surrounding environment (glass substrate and air in our experiments). This equation cannot predict the exact resonance position and does not consider factors such as metal thickness, hole diameter and shape, which also greatly affect the optical response.

Moreover, in periodically perforated ultrathin metal films $\left(<100\;\mathrm{nm}\right)$, excitation of the SPPs results in the suppression of the transmittance rather than amplification, and also a strong enhancement of the absorptivity, in contrast to what is expected for thicker films. This phenomenon is attributed to the coupling between the SPPs at the two metal interfaces, which gives rise to long range (LR) and short range (SR) SPP resonances [69].

Metal NH arrays arranged between two dielectrics of different refractive index, that is, on glass substrates and immersed in air, as in this work, are expected to exhibit a more pronounced SR SPP corresponding to the high wavelength mode, the LR SPP being destroyed almost completely [38]. Thus, the transmission peaks that appears in the spectra in Figure 5b, starting from approximately 800 nm (10 min sample) up to 950 nm (15 min sample) could be assigned to the SR SPP (1,0) resonant peak, while the weaker peaks around 630–650 nm could be assigned to the LR SPP (1,0).

Grating coupling also determines non-plasmonic diffractive features known as Rayleigh-Wood’s anomalies (RWA) that contribute to transmission minima.

The relation for the free-space incident wavelength of an RWA in the case of hexagonal arrays is:

(2) $$\lambda_{min}\left(i,j\right)=\frac{\sqrt{3}}{2}\frac{P}{\sqrt{i^{2}+ij+j^{2}}}\sqrt{\varepsilon_{d}}$$

According to this equation, the $\left(1,0\right)$ RWA minimum at the glass interface should be at 650 nm and could contribute to the dip around 670 nm and 690 nm relative to the samples obtained with 10 min and 12 min O_2_ plasma treatment, respectively. RWA is known to give life to rather weak figures and is often covered by other processes [70]. The cited mode at 650 nm is, in fact, not visible in the other samples, and the $\left(1,1\right)$ at glass interface and the $\left(1,0\right)$ at the air interface, predicted at 459 nm and 433 nm, respectively, are not entirely visible, probably because they are overwhelmed by the intra-band transition peak of gold at 500 nm. Further orders are out of our spectral range.

**Figure 6:**
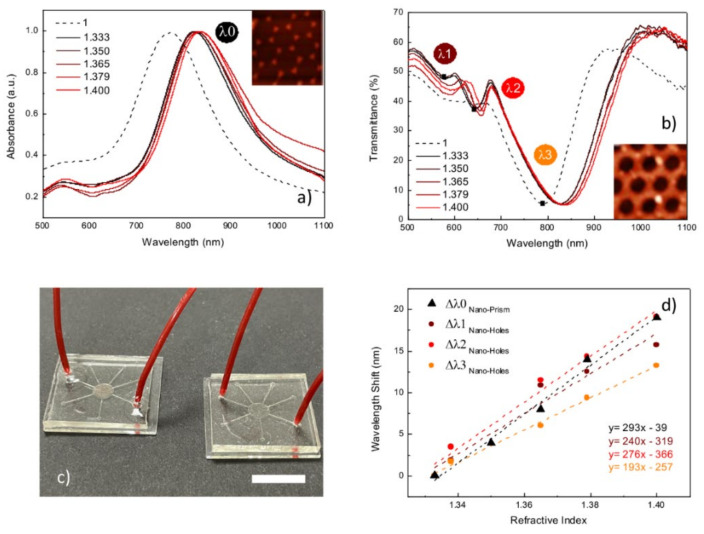
Absorption (**a**) and transmittance (**b**) spectra of Au nanoprisms and nanoholes array with an average diameter of 350 nm, respectively exposed to different glycerol concentrations; both systems were fabricated by NSL; (**c**) picture of the miniaturized lab-on-a-chip (LOC) platform based on the integration of nano-structured LSPR transducers into a 2.5 cm × 2.5 cm PDMS microfluidic chip; scale bar is 1 cm; (**d**) comparison of the calibration curves vs the refractive index calculated for the fabricated systems.

The sensing capabilities of the fabricated nanostructures were experimentally tested by bulk refractometric measurements taken by monitoring optical absorption or transmittance in presence of an increasing refractive index of the external environment. Two different plasmonic materials were exposed to solutions prepared by glycerol diluted in deionized water. In particular, Au nanoprisms and Au NH with a diameter of 350 nm were compared, respectively. The optical responses of the nanostructures were calibrated to specific refractive index (RI) variations in the range from 1.333 to 1.400. By submerging these samples in the prepared solutions, the variations of the LSPR absorption peak (λ_0_) for gold nanoprisms and three different transmittance minima (λ_1_, λ_2_, λ_3_) for the metal nanohole array were monitored. As shown in Figure 6a,b the effect of the high-index dielectric surroundings and of re-adjusting the refractive index symmetry above the top and bottom metal surfaces, gave rise to a spectral shift of the resonances toward longer wavelengths. The calibration curves calculated from the wavelength shift (nm) of the LSPR absorption peak (or transmittance minima) are reported in Figure 6d. The analyzed materials were able to detect even slight variations of the surrounding optical properties. In particular, they exhibited a linear dependence of plasmonic response on the refractive index of the external environment. From the slopes of the calibration curves, a bulk refractive index sensitivity of 293 nm/RIU was estimated for the λ_0_ of the periodic array of the nanoprisms, while three different values were calculated for the periodic array of the nanoholes, respectively 240 nm/RIU for λ_1_, 276 nm/RIU for λ_2_ and 193 nm/RIU for λ_3_. In fact, as already seen, nanohole arrays offer a variety of plasmonic resonances due to the interplay between the localized and propagating modes. This intrinsic property offers several advantages, not only regarding the number of SP resonances available at different spectral positions, but also the possible increase to the electric field enhancement owing to the presence of modes hybridization. [71]

These results demonstrate the possible exploitation of these very cheap plasmonic materials. In fact, the tunability of the nanostructure in terms of morphology and optical properties opens up a range of applications from optical biosensors, to plasmon enhanced fluorescence, to surface Raman enhanced spectroscopy.

## 4. Conclusions

In conclusion, we exploited a nanosphere lithography-based technique for the rapid and highly reproducible fabrication of nano-structured plasmonic material on a very large area. This technique enables the fabrication of a periodic distribution of metal nanoprisms or metal nanohole array starting from a thin metal layer. Similar metamaterials exhibit interesting plasmonic properties which can be precisely modulated in a selected spectral range. The developed technique is based on the modification of a close-packed array of PS nano-spheres after their deposition onto flat substrates. A post-processing oxygen plasma treatment was applied to reduce the dimensions of PS particles, inducing the formation of a non-close packed array of colloids. A simple and effective optical characterization technique was implemented in the fabrication process to precisely control the dry etching process and therefore the morphological and optical properties of the nano-structured materials. This approach enlarges the fabrication capability of conventional nano-sphere lithography, which can only generate triangular-shaped nanoprisms with a predetermined size and offers the potential of tuning the optical response of the fabricated nanostructure to be resonant with different (bio)molecules, making these structures ideal multifunctional plasmonic substrates for biological and chemical sensing.

## Figures and Tables

**Table 1:** The nanosphere dimensions obtained after oxygen plasma treatment, reported and related to the etching duration and the spectral position of the absorbance peak.

Etching Time (min)	Wavelength (nm)	Sphere Diameter (±20 nm)
0	640	500
2	635	470
4	617	440
6	583	420
8	560	400
10	550	380
12	513	350
14	455	200
15	450	185
16	440	150

## Data Availability

Not applicable.
